# Reminiscences of the Insane

**Published:** 1887-12-10

**Authors:** D. Hack Tuke

**Affiliations:** late President Medico-Psychological Association, etc.


### Reminiscences of the Insane.

By D. Hack Tuke, M.D., LL.D., F.R.C.P., Lon. (Hanwell), late President Medico-Psychological Association, etc.

(*Concluded from p.* 97, *vol. ii.*)

**Sir William Ellis.**

In the past history of the treatment of the insane in England there has been some forgetfulness of the services rendered by Sir William Ellis, both at Wakefield and Hanwell. It is easy to explain how this has come about. His work was eclipsed by that of Dr. Conolly; and yet in the matter of non-restraint there was a genuine wish on Sir William's part to reduce the amount as much as possible. In fact, he had great difficulty in lessening the resort to restraint to even the extent to which he achieved success. Our belief is, that notwithstanding his efforts, there would be six or seven per cent. in restraint at the time of which we speak, when he occupied the post of superintendent of Hanwell Asylum; this belief being founded upon conversation with those who worked under him. The restraint-chair, the strait-waistcoat, and muffs were the forms of restraint then employed. He belonged to the Wesleyan body, and possessed the Greek Testament which Wesley used at Oxford. One of his patients may be said to have anticipated the Davenport Brothers. He was an acute maniac, who had been placed for the night in a separate room, and tore up a strong hemp sheet with which he had been provided, into strips about an inch in breadth. In the morning the attendant found him with these strips elaborately wound round his limbs, and it required two hours to remove them. The trouble occasioned by this unwinding had, at any rate, the advantage of affording the patient infinite pleasure.

**Non-Restraint.**

**Patient Anticipates Davenport Brothers.**

**Removal of Chains at York Retreat.**

The good effects of liberating patients from the ingenious restraints by which they are frequently secured are often the most striking. In the early days of the York Retreat, when a strenuous effort was made to appeal wherever possible to what remained of reason in the patient rather than to brute force, a patient's chains were removed on entering the institution, and he was conveyed to the room in which the superintendent and matron were at supper. The novelty of the situation calmed him, especially as he was asked to sit at table with them. After supper he was quietly taken to his bed-room, and assured by the superintendent that he would have every kindness shown to him. The hope was expressed that he would so conduct himself that it would be unnecessary to resort to mechanical restraint. He promised to restrain himself, and although at times vociferous no coercion in the form of personal restraint was ever employed. We have known, however, a somewhat different result.

**Lieut. ——**

Lieut. —— was admitted to the —— Asylum, having been carefully strapped for seven years. He was at once released from his bonds, and on the same day he dined with the family of the superintendent. In a day or two, however, he became so outrageously violent that it required seven men to control him, and, being possessed of great muscular power, he was able to draw them all along the passage in a contrary direction to that which they desired. It sometimes happens that the patient himself perceives the necessity of restraint, as was the case with this powerful lieutenant. He would say when he felt the paroxysm coming on, "If you are friends to yourselves you will secure me." He was fastened with a strap around his waist, and his hands placed in a leathern muff. One day the attendant happened to say that he was suffering from toothache, and that he thought there was nothing in the world worse to bear than that. Lieut. —— exclaimed, "Yes there is," and, moving his muff-secured hands up and down, added, "Here's a melancholy instance of it."

**A Mad Officer.**

In another instance, for want of sufficient care, a patient escaped who had been released from his fetters. This man was a military officer, who one day galloped, sword in hand, into the yard of an inn not far from the —— Asylum. He had escaped from his regiment raving mad, and was slashing his horse wildly with his sword. Having been secured with difficulty he was handcuffed by a constable, who took him in a post-chaise to the barracks, where he arrived with a leg out of each window. The army doctor treated the matter lightly, said he would soon be well, and directed his handcuffs to be removed. The consequence was he shortly afterwards escaped from the barracks, ran over the fields at a tremendous speed, and it was with great difficulty that he was recaptured before doing injury to himself or others. The doctor did not fail to profit by the lesson. It is very proper to grant liberty to madmen, but it must be accompanied by suitable precautions, and a sufficient force at hand, whether visible or not to the patient. Here as elsewhere, although "force is no remedy," it cannot be altogether dispensed with.

**Force Necessary as well as Liberty.**

**Patient Cured by a Ruse.**

It is very true that, as a general rule, it is worse than useless to argue with patients about their delusions or to trick them into recovery. But as all rules have their exceptions, so here. A woman in the —— Asylum was in a state of melancholy depression, tormented with the delusion that she had a May fly in her head. She averred that she heard it buzzing now in one part, now in another, and that it made her utterly miserable. All treatment failed to benefit her. At last the doctor resolved to relieve her of her trouble by an ingenious ruse. He assured her that he would remove the fly, and proceeded to make a slight incision in the scalp. Having taken the precaution to secrete a large fly, he exclaimed that he had captured the enemy which gave her so much annoyance, and showed to the patient the fly he had in his hand. She was delighted with his success, recovered from that moment, and before long left the institution entirely free from her delusion and auditory hallucination. She remained in good mental health for some five years. Then a well-meaning but indiscreet brother informed her by what means she had been cured. Sad to relate, she at once relapsed into her former melancholy state, had precisely the same delusion, and was tormented with the same distressing buzzing. She returned to the Asylum, but the same means of cure could not be again resorted to. The doctor was very indignant with the unwitting cause of the relapse, as well he might be. Bearing on the cure of patients by methods of this kind is the occasional recovery of a patient by means of hypnotic suggestions. It remains to be seen whether this mode of treatment admits of any considerable application, but the undoubted fact that it is sometimes successful in removing a delusion is of great importance from the proof it affords that cases of insanity occur in which there is no great change in the structural components of the brain, but a morbid, mental belief, which by some faulty working of the machine has taken possession of the mind, in consequence of which error assumes the semblance of truth; yet so ideal is the stage, and so superficial is the change in the material organ that a counter-impression derived from a powerful suggestion made in a peculiarly susceptible state of the nerve-cells masters the morbid idea and replaces it with one which is healthy. It is not, however, in the susceptible state of hypnotism only that this form of moral treatment is possible, but the same prin-

**Relapse.**

**Hypnotic Suggestions**

**Their Teaching.**

**Appeals to Reason.**

ciple is involved whenever the physician cures a patient by appeals to his good sense and reason. For example, if a woman labours under the delusion that her child is dead while she is at a distance from it, assurances that it is living may altogether fail, and the mother be plunged into a profound state of melancholy, and yet if the child be brought to her she may be convinced that it is living, and she may recover from that moment. It is therefore always worth while to try the experiment of bringing the patient face to face with the subject of his delusion, and not too readily assume that if one delusion is dispelled, another will take its place. The friends of patients can sometimes render great assistance to the mental physician, in aiding him in the attempt to make a trial of what may be called the reasoning method of treatment as compared with the pharmaceutical, however exceptional the success of this form of treatment may be. Sufficient that it is sometimes successful when all other remedies have failed, and that the resort to it at the proper time requires in many instances that the design of the physician should be united in and assisted by judicious friends.

**Help from Friends.**

All such reminiscences as the foregoing recall a vast number of cases of great variety, both as regards causation, the results of treatment, and the varying degrees of gratitude, or its complete absence, towards those who have been their best friends.

But it is now time to bring them and the reflections which so naturally arise in the retrospect to a conclusion. Amidst a number of thoughts which are suggested, the difficulty is to repress and condense, and to avoid the temptation to prolixity. We could, had we chosen, have dwelt more upon the joyful phases and even the amusing incidents of the insane world; and if the impression produced upon the reader is one of unmitigated sadness, it would be contrary to the fact if not relieved by a very considerable touch of happiness. We have sought to depict some of the successes of sane contests with insane tendencies and developments, and here all will unite in feeling an interest in the result. Such a contest appeals to all. The hard struggle, the battle and the triumph, the Olympian wreath barely won, as the burning wheels dart like lightning to the goal, or the heroic endurance which no success rewards, make up the charm of all history and all romance, and, we maintain, are not wanting in even the "Reminiscences of a Mental Physician."

